# Trends in nonurgent mental health presentations to emergency departments: insights from the first nationwide study in Germany

**DOI:** 10.1186/s12888-025-07368-0

**Published:** 2025-09-10

**Authors:** Heribert Kirchner, Sandra Mangiapane, Emil Hu, Nik Hulsmans, Patrick Brzoska, Frank-Gerald Bernhard Pajonk

**Affiliations:** 1 Faculty of Health, School of Medicine, University Witten/Herdecke, Herrhausen-Straße 50, Witten, 58455 Germany; 2 Central Research Institute of Ambulatory Health Care in Germany (Zi), Salzufer 8, Berlin, 10587 Germany; 3 Department of Psychology, University of Siegen, Adolf-Reichwein-Straße 2a, Siegen, 57076 Germany; 4 Zentrum für seelische Gesundheit, Klinikum Siegen, Weidenauer Str. 76, Siegen, 57076 Germany; 5 Zentrum Isartal Am Kloster Schäftlarn, Schäftlarn, Germany; 6 Department of Psychiatry and Psychotherapy, Technical University Munich, Munich, Germany

**Keywords:** Nonurgent, Mental health emergency, Hospital, Emergency department, Trend

## Abstract

**Background:**

Patients with mental health conditions represent a significant concern in emergency departments, consistently ranking as the third or fourth most prevalent diagnoses during consultations. Globally, over the past two decades, there was a marked increase in such incidences, largely driven by a rise in nonurgent visits related to somatic complaints. However, the implications of these nonurgent visits for mental health patients remain unclear, and warrant further investigation. The aim of this study is to determine the significance of nonurgent mental health patients in emergency departments through pioneering a nationwide dataset collected over a 10-year observation period.

**Methods:**

The study utilized German outpatient billing data at emergency departments of general hospitals (only emergency cases) from 16 Associations of Statutory Health Insurance Physicians (Kassenärztliche Vereinigungen (KVs)), overall covering the years 2012 to 2022, and analyzed in further detail for diagnoses of cannabis (THC-) related disorders (F12.x), depressive Episode (F32.x) and anxiety disorder (F41.x) in the year 2019.

**Results:**

The analyses revealed a notable increase in the number of cases per 100,000 inhabitants over the study period, with an accelerated growth trend observed from 2016 onwards. Pandemic-related effects were evident during 2020–2021, followed by a renewed rise in such incidences during the post-pandemic period.

**Conclusions:**

The increase in nonurgent mental health emergency cases could be influenced by prevalence and demographic changes, shifts in healthcare-seeking behavior, outpatient undersupply and enhanced documentation of psychiatric diagnoses. Consequently, further detailed analysis of the underlying causes and their implications for such evidence is required. In addition to expanding low-threshold services and public education, improved coordination within the regional care system—particularly through a strengthened gatekeeper role of general practitioners—could play a crucial role.

## Background

Global research has highlighted the critical role of mental health emergency (MHE) patients in shaping resource utilization and demand within emergency departments (EDs). Recent studies have offered preliminary insights into the underlying reasons for their presentations, transcending differences in countries and healthcare systems [1–6]. Within Germany, approximately 9.8 million outpatients sought emergency care in EDs during 2021 [5], with MHEs accounting for an estimated 5–10% of these cases [1]. However, the proportion of nonurgent MHE patients within this large cohort remains uncertain due to the limited research available on this specific issue.

Mental health conditions present additional challenges in EDs. The role of nonurgent MHE in exacerbating ED caseloads – linked to overcrowding and reduced treatment quality remains underexplored [3, 4, 7]. While U.S. studies have reported rising cases and diverse psychiatric diagnoses, such as substance-related issues and anxiety disorders [1, 2, 8, 9], European data focus primarily on diagnosis frequency, though without addressing nonurgent cases [8, 9]. Efforts in nations such as Australia and the U.S. have introduced triage systems tailored to psychiatric patients to ensure urgency-based care, akin to protocols for somatic emergencies [10–12].

Conversely, research on low-urgency somato-medical emergency contacts is well-documented [13–15]. Regardless of which ED was investigated, the reasons for nonurgent somato-medical patient visits were very similar throughout [16–18]. Patients sought ED treatment due to their subjectively perceived exceptional health situation. The main reasons included long waiting times for appointments with specialists, together with low-threshold and the prospect of competent 24/7 services offered by the local ED. Interestingly, a significant proportion of nonurgent patients still considered themselves to require emergency treatment [9, 18]. Frequently changing, less stable relationships with a primary care physician were also cited as a contributing factor for patients to attend the ED [8].

The German outpatient healthcare system plays a crucial role in providing primary and specialized care. General practitioners (GPs) or family physicians (“Hausärzte”) are the primary point of contact for most patients, coordinating their healthcare needs. While patients can directly access specialists or EDs without a GP referral, many still prefer consulting their GP as the first port-of-call. Increasing ED utilization is often linked to a shortage of GPs and specialists, particularly in rural areas, together with long waiting times for appointments [19]. Younger, healthier individuals without a regular GP and patients seeking more rapid access to care frequently seek to utilize EDs. This trend has grown over the past decade and is compounded by a substantial shortage of psychiatrists in Germany.

The aim of this study was to evaluate the relevance of nonurgent mental health patients in EDs by leveraging, for the first time, a comprehensive nationwide dataset spanning a 10-year observation period. This approach allowed for an in-depth analysis of trends, patient characteristics, and healthcare utilization patterns associated with nonurgent mental health cases. By examining such data, this study sought to provide novel insights into the burden such as patients demographics places upon EDs, including impact on healthcare resources, and potential implications for care delivery and policy development.

## Methods

In 2024, we conducted a retrospective analysis of pseudonymized nationwide outpatient billing data provided by 16 Associations of Statutory Health Insurance Physicians (Kassenärztliche Vereinigungen, KVs) in Germany. The study period covered 2012 to 2022 and included all patients who presented to a general hospital emergency department (ED) with at least one mental health diagnosis (ICD-10 F0–F9). In the German healthcare system, psychiatric emergency patients not admitted as inpatients are billed through the Associations of Statutory Health Insurance Physicians under the outpatient system rather than the hospital-specific DRG system. However, the use of an emergency billing code allows for identifying these patients as emergency cases, albeit categorized as “nonurgent mental health emergency patients” according to the German S2k-Guideline for Emergency Psychiatry (2019).

Based on the assumption that emergency contacts without subsequent inpatient admission are to be categorised as remaining outpatient cases, it was concluded that these cases did not fulfil the criteria of a psychiatric emergency. As no individual case-by-case examination of the billed emergency contacts was carried out, this approach is an approximation in the sense of an epidemiological estimate.

A two-step approach was employed:

1. Initial Inclusion and Trend Analysis (2012–2022): All patients with at least one primary or secondary mental health diagnosis who visited an ED were included to examine overall case numbers and trends over the 10-year period. This broad inclusion criterion encompassed both patients with a single mental health diagnosis and those with comorbidities.
2. Focused Analysis of Selected Diagnoses (2019): In the second step, we narrowed our focus to three specific ICD-10 categories — F12 (mental and behavioral disorders due to psychoactive substance use, especially cannabis (Tetrahydrocannabinol, THC), F32 (depressive episodes), and F41 (anxiety disorders) — to gain more detailed insights. We analyzed data from 2019 and examined patient age, sex, and the presence of a single mental health diagnosis. All ED visits during this year were included, regardless of whether they occurred during or outside regular office hours. The selection of the three diagnoses from the main categories substance use disorders, affective disorders, and neurotic, stress and somatoform disorders for in-depth analysis were chosen for the following reasons: detail below. The depressive episode (ICD-10 F32.x) was chosen because it is highly likely to represent a first episode of an affective disorder, and the emergency department (ED) may serve as an initial point of contact for affected patients. The THC-associated disorders (ICD-10 F12.x) were included in the analysis to assess their relevance in emergency care over the past decade. Furthermore, their selection was motivated by the legislative change in Germany in April 2024 regarding the legalization of cannabis. For the first time, the prevalence of anxiety and panic disorders was specifically examined in this scale in the setting of an ED. This decision was based on the generally high prevalence of these disorders, while no reliable data on their occurrence in emergency medicine have been available to date. This study represents an initial step toward assessing the relevance of nonurgent psychiatric patients in German EDs. A follow-up study with a broader scope is already planned. This methodology enabled us to first capture a comprehensive overview of mental health presentations in EDs over time and then to examine key diagnostic groups more closely, providing valuable insights into patient characteristics and service utilization patterns.

### Statistical methods

To explore the development of case and patient numbers, rates per 100,000 inhabitants were calculated to ensure better comparability. Regarding the analysis of age distribution, age-standardized values are reported. For descriptive analysis, the data were presented using graphical representations and tables.

The overall trend in case/patient numbers (AV) was analyzed using regression analysis, with time (in years) included as an independent variable in the model. For the specific analyses of individual diagnostic groups (F12, F32, and F41 according to ICD-10), the development of diagnosis-specific case/patient numbers was initially examined using the same regression analysis approach. If the assumption of homoscedasticity was violated, robust standard errors were calculated according to Hayes and Cai [20]. Moderate violations of the normality assumption were tolerated due to the robustness of the analysis method (Schmidt & Finan, 2018). Additionally, the data were analyzed for age- and gender-specific differences in the distribution of case/patient numbers. Due to the categorical nature of the data, the corresponding analyses were conducted using Chi-square tests. If a cell frequency of < 5 was present, the calculation was performed based on a Monte Carlo simulation with 5,000 replications. To prevent alpha error accumulation in pairwise comparisons, the Bonferroni correction was applied. A conventional significance level of ⍺ < 0.05 was used to determine statistical significance.

In our analysis, we chose to use linear regression as the primary method, as it is widely applied in the relevant literature and often allows for a more accessible interpretation. While we are aware of the methodological limitations associated with this approach, we conducted a Poisson regression as a sensitivity analysis to ensure the robustness of our findings. The core results remained consistent across both modelling approaches. Furthermore, model fit was assessed using the AIC and BIC criteria, which showed only minor differences between the models (AIC linear: 93.67 vs. Poisson: 94.22; BIC linear: 94.86 vs. Poisson: 95.02).

## Results

### Mean proportion of psychiatric presentations among all outpatient ED visits

Nationwide case figures on the utilisation of emergency care in Germany are available, among others, for the period from 2009 to 2019 [21]. These include various emergency situations depending on the place of care and the patient’s further stay in the care system. On average, around 9.36 million outpatient emergency contacts were documented annually in the ED during this period. In addition, an annual average of around 7.66 million cases were admitted to hospital as inpatients in an emergency, preceded by a presentation in the ED (DRG-associated cases with emergency as the reason for admission). In addition, around 8.52 million emergency patients were treated annually as part of the medical on-call service or in emergency practices of the Associations of Statutory Health Insurance Physicians („Notfallpraxis der Kassenärztlichen Vereinigung“). The average annual number of cases per 100,000 inhabitants between 2009 and 2021 was approximately 11,420 for outpatient presentations to emergency departments and around 9,342 for emergency department contacts resulting in subsequent inpatient admission.

The average proportion of outpatient emergency contacts with a psychiatric primary or secondary diagnosis was 239,913 cases per year in the period from 2012 to 2019. This corresponds to an estimated share of 2.58% of all outpatient emergency contacts (basis: average of 9.31 million cases per year).

**Fig. 1:**
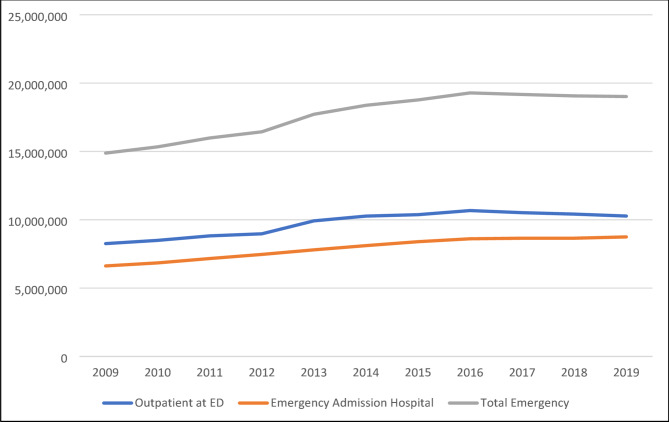
Trend in Outpatient ED Visits and Emergency Admissions at the Hospital

As illustrated in Fig. 1, the number of outpatient cases increased steadily until peaking in 2016, followed by a continuous decline thereafter. In contrast, the proportion of psychiatric outpatient presentations among all emergency department (ED) visits in Germany rose consistently during this period—from 2.1% to over 3.0%. Moreover, the total number of emergency hospital admissions showed a continuous upward trend throughout the observation period.

### General trend of case numbers with primary and/or secondary mental health diagnosis

**Fig. 2:**
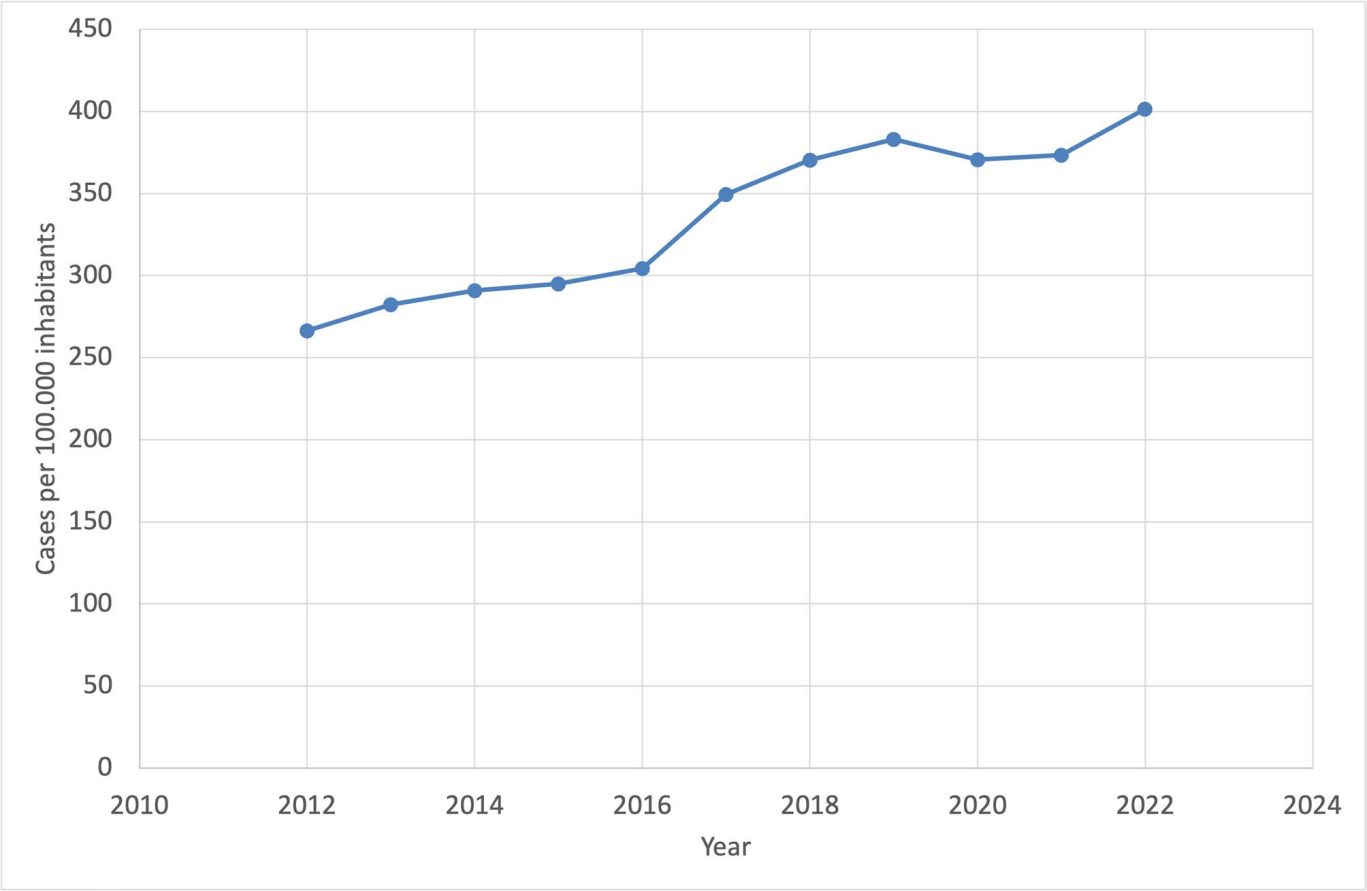
General trend in case numbers from 2012 to 2022 with primary and/or secondary mental health diagnosis

In 2012, the rate of outpatient emergencies due to mental health diagnosis was 266.6 cases per 100,000 inhabitants, rising to 401.5 cases by 2022—a substantial increase of 50.7% over the 10-year period. Between 2012 and 2016, the growth was relatively moderate, with the rate increasing from 266.6 to 304.4 cases per 100,000 inhabitants (+ 14% over four years). However, from 2016 onward, the rate accelerated notably, particularly between 2017 and 2019 (see Fig. 2), when cases rose from 349.5 to 383.0 (+ 9.6% over two years). A regression analysis shows that the rate (2012–2022) of primary and/or secondary mental health diagnosis increases by about 13.83 per 100.000 inhabitants annually.

The analysis reveals that the annual increase in cases per 100,000 inhabitants has risen over the past 10 years, indicating a growing prevalence of the condition within the population. During the COVID-19 pandemic (2020–2021), growth stagnated or slightly declined. In 2020, the rate dropped to 370.7 cases (−3.2% compared to 2019), followed by a minimal increase to 373.5 cases in 2021 (+ 0.8% compared to 2020). In 2022, the upward trend resumed strongly, with the rate reaching 401.5 cases per 100,000 inhabitants (+ 7.5% compared to 2021).

### Trends in emergency presentations for F12.x, F32, and F41 diagnoses from 2012 to 2022

Building upon the initial assessment of temporal trends in psychiatric emergency presentations, a diagnosis-specific analysis was conducted for F12.x (cannabis-related disorders), F32 (depressive episodes), and F41 (anxiety disorders). The development of these three diagnostic categories over the period from 2012 to 2022 reveals distinct trajectories in terms of prevalence, temporal dynamics, and regression characteristics.

#### F12.x (cannabis-related presentation)

In 2012, the nationwide rate of emergency department (ED) presentations involving a primary and/or secondary F12.x diagnosis was 2.72 cases per 100,000 inhabitants. By 2022, this rate had more than tripled, reaching 8.97 cases per 100,000 (see Table 1). The average annual percentage increase was 13.2%, indicating a substantial and sustained rise in presentations over the observation period. Regression analysis confirmed a highly significant linear trend for both the number of cases (b = 5.87, SE = 4.13, *p* <.001, R²_adj = 0.97) and the number of unique patients (b = 5.46, SE = 3.90, *p* <.001, R²_adj = 0.97). After correcting for the violation of the homoscedasticity assumption, time (2012–2022) remained a significant explanatory factor for case numbers (b = 0.69, SE = 0.05, *p* <.001, R²_adj = 0.97), confirming the robustness of the trend.

#### F32 (depressive episodes)

Depressive episodes were frequent psychiatric diagnoses in EDs during the study period. The incidence of F32 diagnoses rose steadily until peaking in 2017, with 26.6 cases per 100,000 inhabitants. A transient decline was observed during the early phase of the COVID-19 pandemic (2020), followed by a return to near pre-pandemic levels by 2022 with 25.6 cases, per 100,000). After correcting for the violation of the homoscedasticity assumption, regression analyses did not show a statistically significant linear change in cases from 2012 to 2022, indicating that the long-term trend for F32 diagnoses was overall stable.

#### F41 (anxiety disorders)

For F41 diagnoses, a continuous and statistically significant increase in case numbers was observed between 2012 and 2022. Rates rose from 3.85 to 13.66 cases per 100,000 inhabitants. Regression analysis—corrected for heteroscedasticity—showed a strong linear increase for case numbers (b = 2.88, SE = 0.16, *p* <.001, R²_adj = 0.98).

While all three diagnostic categories showed upward trends over the 11-year period, the steepest relative increase was observed in cannabis-related (F12.x) presentations, though absolute rates remained lower than for depressive (F32) and anxiety disorders (F41). F41 demonstrated a steady and robust increase in cases.

### Specific analysis of all three presentations at EDs from 2012 to 2022

**Table 1:** Specific analyses of primary and/or secondary associated ED presentations from 2012 to 2022 (per 100,000 Inhabitants)

Year	Case F12.x	Increase Case (%) F12.x	Case F32.x	Increase Case (%) F32.x	Case F41.x	Increase Case (%) F41.x
2012	2.72	-	22.27	-	39.44	-
2013	3.34	22.57	23.51	5.58	43.78	11.01
2014	3.96	18.73	24.57	4.5	47.41	8.29
2015	4.84	22.18	24.49	−0.3	49.66	4.74
2016	5.28	8.99	24.53	0.13	52.36	5.43
2017	6.63	25.65	26.66	8.7	56.88	8.63
2018	7.24	9.15	25.59	−4	59.47	4.46
2019	8.32	14.97	25.86	1.06	63.22	6.42
2020	8.61	3.5	23.38	−9.6	63.55	0.51
2021	8.73	1.35	24.49	4.75	65.97	3.81
2022	8.97	2.77	25.68	4.86	68.44	3.74

### Age distribution of F12.x, F32.x and F41.x monodiagnoses in 2019

As outlined in the methodology section, cases with a primary and/or secondary diagnosis in the ICD-10 categories F12, F32, and F41 were identified in the second step of the 2019 data analysis. The focus was on cases with monodiagnoses, which were analyzed with respect to age and gender distribution.

#### F12.x (cannabis-related disorders)

Among 6,572 patients presenting with cannabis-related issues in 2019, a total of 5,712 individuals (86.9%) were documented with a single diagnosis (monodiagnosis). Age data were available for 1,447 patients (25.3% of the monodiagnosis group). The distribution of age groups did not significantly differ from the expected population-based distribution. This was confirmed by Chi-square testing for both age-standardized case numbers (χ², *p* =.10) and patient numbers (χ², *p* =.29). The available data predominantly represented individuals under 45 years of age; no valid age-specific data were available beyond the age group 45–49. Values below 30 were standardized to 29 for statistical processing.

#### F32.x (depressive episodes)

For F32, 21,511 cases were recorded, of which 21,477 contained an age indication. 7,082 cases had a monodiagnosis, for all of which the gender assignment was also available. In the F32.x monodiagnosis sample for 2019, the majority of cases were observed in patients aged 20 to 40 years. Despite this concentration, no statistically significant deviation from the expected age distribution was observed. The Chi-square test yielded non-significant results for both age-standardized case numbers (*p* =.11) and patient numbers (*p* =.19), indicating an age pattern broadly in line with the general population structure (see Fig. 2).

#### F41.x (anxiety disorders)

A total of 52,582 cases were identified for F41, of which 52,580 were age-determined. There were 16,253 monodiagnoses with complete information on age and gender. In contrast to the other two diagnostic categories, the age distribution of F41.x monodiagnoses deviated significantly from the expected pattern. The majority of patients were again between 20 and 40 years of age. However, chi-square tests showed significant differences for age-standardized case numbers (*p* <.001), suggesting an overrepresentation of younger adults in this diagnostic group relative to population norms.

**Fig. 3:**
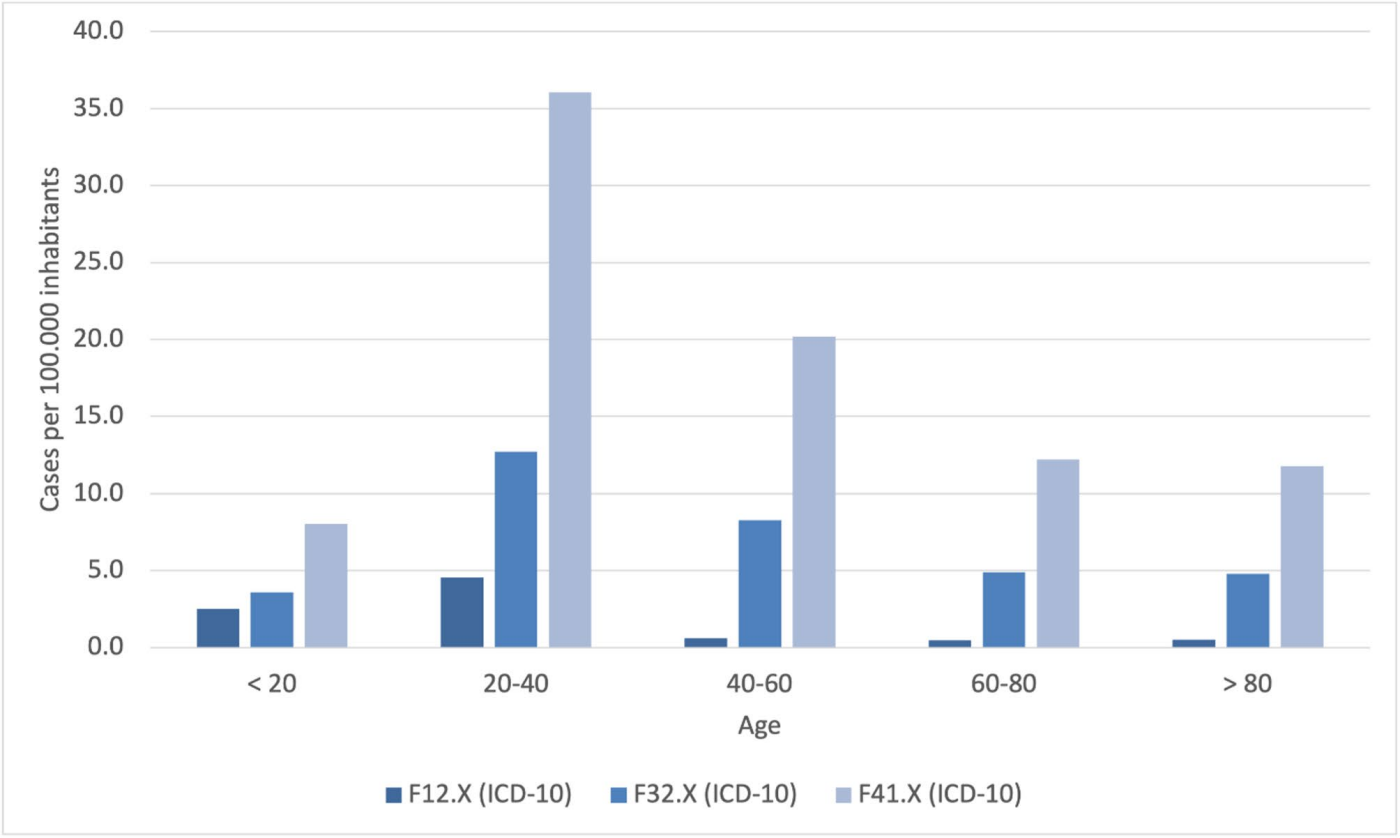
Age Distribution of Psychiatric Diagnoses (F12, F32, F41) in 2019

While all three diagnostic groups showed a concentration of cases among individuals aged 20 to 40 (see Fig. 3), only the anxiety disorder group (F41.x) demonstrated a statistically significant deviation from the expected age distribution. In contrast, the distributions for both cannabis-related disorders (F12.x) and depressive episodes (F32.x) did not significantly differ from population-based expectations.

### Sex distribution of F12.x, F32.x and F41.x monodiagnoses in 2019

To investigate potential sex-related differences in the presentation of psychiatric emergency patients, we analyzed the gender distribution of monodiagnoses in the ICD-10 categories F12.x (cannabis-related disorders), F32.x (depressive episodes), and F41.x (anxiety disorders) for the year 2019, standardized per 100,000 inhabitants.

#### F12.x (cannabis-related disorders)

Among patients presenting with a single F12.x diagnosis, no significant sex-based differences were observed. The Chi-square test yielded non-significant results for both case numbers (*p* = 1.00) and patient numbers (*p* = 1.00) per 100,000 inhabitants, indicating a gender distribution consistent with general population expectations.

#### F32.x (depressive episodes)

Similarly, for patients diagnosed with F32.x as a monodiagnosis, the Chi-square analysis showed no statistically significant deviation in the observed gender distribution. Both case rates (*p* = 1.00) and patient rates (*p* = 1.00) per 100,000 inhabitants reflected proportions expected based on the general population structure.

#### F41.x (anxiety disorders)

For F41.x monodiagnoses, the gender distribution also did not significantly deviate from population norms. The Chi-square test revealed non-significant results for both case numbers (*p* =.50) and patient numbers (*p* = 1.00) per 100,000 inhabitants.

Across all three diagnostic groups, no statistically significant sex-related differences were found in emergency department presentations in 2019. The observed distributions of cases and patients per 100,000 inhabitants were consistent with gender proportions in the general population.

## Discussion

The objective of this study was to examine the proportion of nonurgent mental health patients presenting to EDs at general hospitals throughout Germany. This is the first nationwide investigation of its kind in Germany. The findings suggest that the frequency of nonurgent psychiatric presentations in EDs could have been underestimated to date.

### Average proportion of outpatient mental health emergency presentations among all outpatient ED visits

The continuous rise in the proportion of psychiatric presentations among all ED contacts in Germany—from 2.1% in 2012 to over 3.0% in 2021—despite a declining overall ED utilisation since 2017, highlights a growing and disproportionate demand for acute mental health services. While approximately 9.36 million outpatient ED cases (on average) and 7.66 million inpatient emergency admissions (on average) were documented annually during this period, psychiatric cases accounted for an estimated 2.58% of all outpatient ED contacts between 2012 and 2019 (239,913 cases per year on average). These trends may reflect insufficient accessibility of outpatient mental health care, evolving help-seeking behaviors, or increased psychiatric morbidity in the population [22, 23]. The stable rise in psychiatric ED utilization—despite structural reforms in emergency care and the temporary effects of the COVID-19 pandemic—emphasizes the need for integrated, diagnosis-specific approaches to acute psychiatric care, particularly in low-threshold and pre-hospital settings [24].

### General trend of MHE incidences with primary and/or secondary mental health diagnosis

When examining the overall trend in outpatient psychiatric emergency patients presenting with a primary and/or secondary diagnosis at the ED of a general hospital - throughout Germany, and over the entire investigated observation period - this study identified several potential developments that must be viewed in a broader context.

Since 2012, the rate at which psychiatric patients presented to the ED increased markedly. One possible explanation is a genuine rise in the prevalence of mental health conditions. Another possible justification is that such patients are increasingly seeking care - primarily through the ED, rather than through other healthcare pathways. Presently, however, the underlying cause remains unclear since insufficient data exists to determine which factors drive this trend. A previous study by the authors (2023) suggested that up to one-fith of all psychiatric emergency visits in Germany could be nonurgent [25]. Similar trends in somatic presentations are well documented internationally [4, 26, 27] and were additionally observed across several German studies [14, 15, 28]. For example a qualitative study by Schmiedhofer et al. (2017) explored the motivations of patients with non-urgent conditions for presenting to emergency departments (EDs) in Germany [15]. The study identified several key factors influencing this behavior. Patients often cited difficulties in securing timely appointments with general practitioners or specialists, leading them to seek immediate care in EDs. The 24/7 availability of EDs provided a level of time flexibility not offered by regular outpatient services. Additionally, patients perceived EDs, particularly those in university hospitals, as offering higher-quality care and access to multidisciplinary services in a single visit. This perception contributed to a preference for EDs over traditional outpatient settings (GP respectively „Hausarzt“). The study also noted that the ease of access to EDs sometimes resulted in patients making less effort to obtain appointments in the outpatient sector. These findings suggest that EDs serve an independent role in outpatient care, and that even with improved outpatient service availability, ED utilization for non-urgent conditions may persist.

During the COVID-19 pandemic in 2020 and 2021, this study’s findings revealed a notable leveling-off, and even a decline, in ED presentations, consistent with global reports [29–33]. The absence of nonurgent psychiatric emergency patients, often with affective or anxiety disorders, could reflect pandemic-related factors. By 2022, however, case numbers rose again, indicating renewed ED utilization. A critical issue is that ED personnel resources have not necessarily increased at the same pace, as previously discussed by the authors [34].

Finally, the general trend of rising incidences also appeared in the 2024 study by Thom et al., which examined the prevalence of ambulatory psychiatric diagnoses in Germany during 2012–2022. The proportion of individuals with outpatient mental disorder diagnoses rose from 33.4 to 37.9%, equating to a + 13.4% increase [35]. Depression remained as the predominant diagnosis, while anxiety disorders and substance-related disorders had greater relative growth. Thom et al. examined national data across all levels of outpatient care, and found that approximately 33% of patients had a psychiatric diagnosis. The study population within this investigation represents a subgroup of these findings, focusing on emergency care, and highlighting the growing utilization of ED resources by psychiatric patients.

### Specific analysis of F12.x presentations at EDs in 2019

Substance-related presentations are among the most frequent causes of psychiatric emergency contacts worldwide, with alcohol-related conditions traditionally being predominant [36, 37]. Given novel German legislation (effective April 1, 2024) and the impending legalization of cannabis, this study focused on THC-related contacts. Across the 2012–2022 period, there was an annual increase of 13.2% (*p* <.00001) in primary or secondary THC presentations, aligning with global data [38–41]. In a subsequent analysis restricted to 2019 THC monodiagnoses 86.9% received only a THC-related ICD-10 F12.x diagnosis, while most emergencies occurred in patients between the ages of 15 and 29 years, and dropping markedly from age 35 years or above. Although there was a trend toward higher case numbers up to age 20–24, age parameter did not significantly explain this study’s findings, contrasting several global results [38, 41, 42].The majority were younger patients, and no significant gender differences emerged, contrary to other recent studies [38, 40, 41].

Overall, this study indicate that THC-related emergency contacts primarily affect adolescents and young adults. Preventive measures should therefore be specifically targeted at these age groups. EDs thus play a key role in both public health and prevention. Whether, and to what extent, these rates will continue to rise following the legalization of cannabis remains a subject of ongoing scientific discussion [39].

### Specific analysis of F32.x presentations at EDs in 2019

Affective disorders are highly relevant in EDs, particularly regarding suicidal tendencies in severe depressive syndromes [43–45]. According to the World Health Organization (WHO), approximately 322 million people worldwide are affected (> 4.4% of the global population), marking an 18% increase in the last decade alone [46]. Concerning Germany, the WHO estimates over four million people suffer from depression, although global data on milder depressive syndromes in ED outpatients remain scarce [25, 47]. This study therefore analyzed first-time depressive episodes (F32) presenting in the ED, assuming minimal coding imprecision.

Between 2012 and 2022, F32 diagnoses (primary and/or secondary) do not significantly explain case number trends, yet they indicate a growing burden of depressive episodes. Though this study`s results are not statistically significant, they align with other studies emphasizing the ED’s role in psychiatric presentations [2, 3, 48]. Parkmann et al. discuss “pull” factors (e.g., accessibility) and “push” factors (e.g., perceived need) that drive ED utilization [49]. Overall growth was steady, with only a slight dip around 2020, likely reflecting pandemic-related disruptions [29, 32, 33], before continuing its upward trend.

One plausible explanation is an actual increase in the prevalence of mental health conditions. Alternatively, individuals with these conditions may be increasingly opting to seek care primarily through the ED, rather than through other healthcare pathways. However, due to limited data, it remains unclear which factors are primarily driving this trend. During 2019, across 21,511 F32 cases, 7,082 (32.9%) were single F32.x diagnoses, indicating that approximately 33% were primarily depressive episodes. Most occurred in younger adults, peaking at ages 20–24, whilethe 15–34-year age group comprised ~ 65% of cases, aligning with previous studies [46–48]. These elevated incidence rates could reflect social stressors, life transitions, or increased mental health awareness [50–52]. Beyond the age of 25 years, case numbers dropped, potentially suggesting under-diagnosis or competing health concerns. Females predominated males in case incidences by over 23%—though without statistical significance, consequently contradicting broader literature pointing to such higher female prevalence [52, 53].

### Specific analysis of F41.x presentations at EDs in 2019

Anxiety disorders significantly impact both routine outpatient care and emergency services, with 4.4% of the global population affected in 2021 [54]. In Germany, 4.74% of the population had a one-year prevalence of anxiety disorders in 2022, while Thom et al. (2024) reported 6.7% in outpatient settings—placing anxiety disorders third among mental illnesses, after affective and substance use disorders [35]. These findings underscore a growing burden on healthcare systems, yet research on nonurgent anxiety ED patients remains scarce. One study from this group [55] aligns with Thom (2024), confirming F41.x among the top three outpatient mental disorders [35]. In this goup´s sample, F41.x was most prevalent, surpassing F12.x and F32.x. Notably, in 2019, across 38,713 patients being studied, 38% with an F41.x diagnosis presented with a monodiagnosis, reflecting earlier work by Marchesi and Biancosino [47, 56]. A statistically significant 5.7% annual growth in F41.x cases from 2012 to 2022 underscores the need for targeted prevention and effective treatments. Contrasting to Thom et al. [35], EDs in Germany appear to be a primary access point for anxiety disorders, which aligns with this goup´s 2023 results [25].

This study´s data also demonstrated that emergency cases typically peak among younger adults (up to 24 years) prior to declining with age. Adolescents (15–19 years of age) presented a marked increase, echoing Boyer et al. and Benarous et al. [57, 58] investigations on heightened risk during transitional phases. The ED may thus serve as a first point of contact for anxiety or panic attacks. Regarding gender distribution, 5,682 males (in comparison to 8,870 females) aligns with broader global trends. Overall, these latest underscores the ED’s crucial role in acute care and secondary prevention for individuals with anxiety, especially those seeking help for the first time.

Various causes for the increase in nonurgent emergency contacts are described in the literature, some of which are also transferable to mental health patients. Many of the factors discussed are also considered possible starting points for countermeasures.

The only study in Germany to date examing the nonurgent mental health presentation at the ED addressing this issue, highlights an insufficient availability of outpatient psychiatric services [25]. In the catchment area of the ED examined, only a few psychiatric practices were available for approximately 250,000 to 300,000 inhabitants. One proposed solution was a coordinated system of open consultation hours across existing practices, without the need for appointments, to improve access for individuals experiencing mental health crises.

Targeted public education to enhance mental health literacy—such as clarifying what constitutes a psychiatric emergency—also appears beneficial. This could take the form of awareness campaigns, similar to previous initiatives focused on depression and suicidal behavior [59].

In addition to expanding low-threshold services and increasing public awareness, improved coordination within the regional healthcare system may offer further benefits. Strengthening the gatekeeping role of general practitioners, combined with clear pathways between emergency departments, specialist clinics, and outpatient providers, would be a crucial element in this effort.

### Limitations

This study provides, for the first time, a nationwide overview of ambulatory psychiatric ED patient cases, over a 10-year observation period. The use of billing codes (Notfallziffer) allowed for clear identification of ED contacts. However, uncertainties arose from its retrospective design, particularly regarding the classification of primary and/or secondary diagnoses, which did not always allow for unambiguous determination of the main reason for ED presentation. Emergency contacts without subsequent inpatient admission were classified as outpatient cases and, based on this operational definition, were assumed not to meet the criteria of a psychiatric emergency. As no individual case validation was conducted, this approach constitutes an approximation and should be interpreted within the limitations of aggregate data analysis. To enhance diagnostic specificity, the authors conducted a more detailed analysis of individual diagnoses. Although only a subset of patients with the three primary diagnoses (F12.x, F32.x, and F41.x) were captured with data on age and gender, the authors firmly believe such findings still provide meaningful insights. Furthermore, coding practices could have varied across institutions and over time, potentially impacting the accuracy of both primary and secondary diagnoses. This study was also unable to account for factors such as comorbidity severity, which could influence presentation patterns. Lastly, gathered data did not capture broader sociodemographic variables that could further elucidate the context of psychiatric emergencies in this setting. Furthermore, only patients with statutory health insurance were included on a nationwide level. Patients with private health insurance could not be included. In addition, no comparison was performed against standard (or routine) care, so it remains uncertain whether the observed increase reflects a higher prevalence rate, or an actual rise in service utilization.

## Conclusions

This study provides, for the first time, nationwide data across an entire decade, concerning nonurgent ambulatory psychiatric emergency patient cases in an ED setting. A more detailed analysis of the three primary diagnoses (F12.x, F32.x, and F41.x) yielded several key insights.

The ED frequently serves as the first point of contact, especially for younger patients, with several age-related fluctuations, depending on the specific diagnosis. Within the scrutinised healthcare setting, notable sex-specific trends emerged, namely, male patients were more likely to present with F12.x diagnoses, whereas female patients more frequently presented with F32.x and F41.x diagnoses.

Across the entire 11-year observation period, there was a general trend of increased ED utilization by patients with psychiatric primary and/or secondary diagnoses, confirmed by in-depth analyses of the three main diagnostic groups.

The COVID-19 pandemic affected case numbers substantially, leading to a marked decline. However, in 2022—following the relaxation of pandemic-related restrictions—there was a renewed and significant rise, reaching novel peak levels that underscore the pressure placed upon German ED services.

Overall, the ED functions as a critical interface between pre-hospital and inpatient psychiatric emergency care, increasingly utilized by a previously under-recognized segment of nonurgent psychiatric patients, while also offering substantial potential for preventive interventions.

## Data Availability

The datasets used are publicly available on request (https://www.zi.de) and can also be requested from the corresponding author.

## References

[1] Larkin GL, Claassen CA, et al. Trends in U.S. Emergency department visits for mental health conditions, 1992 to 2001. PSYCHIATRIC Serv June. 2005;56:672–7. 10.1176/appi.ps.56.6.671 15939942

[2] Hazlett SB. Epidemiology of adult psychiatric visits to U.S. Emergency departments. Acad Emerg Med. 2004;11:193–5. 14759965

[3] Capp R, Hardy R, Lindrooth R et al. National trends in emergency department visits by adults with mental health disorders. J Emerg Med 2016; 51: 131–e1351. 27614303 10.1016/j.jemermed.2016.05.002

[4] Hooker EA, Mallow PJ, Oglesby MM. Characteristics and trends of emergency department visits in the united States (2010–2014). J Emerg Med 2019; 56: 344–51. 30704822 10.1016/j.jemermed.2018.12.025

[5] Statista. 13.01. Weltbevölkerung mit Depression nach Geschlecht | Statista (2025). Im Internet: https://de.statista.com/statistik/daten/studie/1078802/umfrage/anteil-der-weltbevoelkerung-mit-depression-nach-geschlecht/; Stand: 13.01.2025.

[6] Statistisches Bundesamt. 9,8 millionen behandlungen in notfallambulanzen Im Jahr 2021 (20.12.2022). Im Internet: https://www.destatis.de/DE/Presse/Pressemitteilungen/Zahl-der-Woche/2022/PD22_51_p002.htmlStand: 14.01.2025.

[7] 7. Duggan M,Harris B, Chislett WK, Calder R. Nowhere else to go: WhyAustralia's health system results in people with mental illness getting ‘stuck’in emergency departments. Discussion Paper,Mitchell Institute, Victoria University, Melbourne; 2020. ISBN: 978‑0‑6488001‑1‑821. Mangiapane S, Czihal T, vonStillfried D. Entwicklung der ambulanten Notfallversorgung in Deutschland von2009 bis 2020. Zi-Paper 16/2021. Berlin: Zentralinstitut für diekassenärztliche Versorgung; 2021.38. Hoch E, Olderbak S, Schwarzkopf L, Gomes de Matos E, Schneider F.Cannabis – Zahlen und Fakten. In: Deutsche Hauptstelle für Suchtfragen (Hrsg.),DHS Jahrbuch Sucht 2024, S. 87–105. Lengerich: Pabst Science Publishers; 2024. ISBN:978-3-95853-910-5.42. Deutsche Hauptstelle fürSuchtfragen (Hrsg.). DHS Jahrbuch Sucht 2024. 1. Aufl. Lengerich: Pabst SciencePublishers; 2024. ISBN 978-3-95853-910-5.

[8] Freudenmann RW, Espe J, Lang D et al. Psychiatrische notfälle auf der medizinischen Notaufnahme des universitätsklinikums Ulm in Den Jahren 2000 und 2010. Psychiatr Prax 2017; 44: 29–35. 26158716 10.1055/s-0035-1552681

[9] Schlump C, Thom J, Boender TS et al. Nutzung von routinedaten Aus Notaufnahmen Zur surveillance von Suizidversuchen und psychiatrischen notfällen. Bundesgesundheitsbl 2022; 65: 30–9. 10.1007/s00103-021-03467-x PMC8661829 34889967

[10] Clarke DE, Boyce-Gaudreau K, Sanderson A, et al. ED triage Decision-Making with mental health presentations: A think aloud study. J Emerg Nurs. 2015;41:496–502. 26033786 10.1016/j.jen.2015.04.016

[11] Smart D, Pollard C, Walpole B. Mental health triage in emergency medicine. Aust N Z J Psychiatry 1999; 33: 57–66; discussion 67 – 9. 10197886 10.1046/j.1440-1614.1999.00515.x

[12] Broadbent M, Moxham L, Dwyer T. Implications of the emergency department triage environment on triage practice for clients with a mental illness at triage in an Australian context. Australasian Emerg Nurs Journal: AENJ. 2014;17:23–9. 10.1016/j.aenj.2013.11.002 24507180

[13] Schmiedhofer MH, Searle J, Slagman A, et al. Inanspruchnahme zentraler notaufnahmen: qualitative Erhebung der motivation von Patientinnen und patienten Mit nichtdringlichem behandlungsbedarf. Gesundheitswesen (Bundesverband Der Arzte Des Offentlichen Gesundheitsdienstes (Germany)). 2017;79:835–44. 27104309 10.1055/s-0042-100729

[14] Somasundaram R, Geissler A, Leidel BA et al. Beweggründe für die Inanspruchnahme von Notaufnahmen – Ergebnisse einer Patientenbefragung. Gesundheitswesen (Bundesverband der Arzte des Offentlichen Gesundheitsdienstes(Germany)). 2018; 80: 621–627. 10.1055/s-0042-112459 27611882

[15] Schmiedhofer M, Searle J, Slagman A et al. Perception of the emergency department for outpatient care in a rural region in Saxony-Anhalt: a qualitative survey of patients and general practitioners. Dtsch Med Wochenschr (1946) 29 Mar 2017: e61–73. 10.1055/s-0043-100639 28355651

[16] Deutscher ÄrzteverlagGH., *Redaktion Deutsches Ärzteblatt*. Zustände in den Notaufnahmen sind „erbärmlich (27.04.2022). Im Internet: https://www.aerzteblatt.de/nachrichten/106908/Zustaende-in-den-Notaufnahmen-sind-erbaermlich; Stand: 27.04.2022.

[17] *Deutscher Ärzteverlag GmbH, Redaktion Deutsches Ärzteblatt*. Inanspruchnahme der ambulanten Notfallversorgung und der ungeplanten stationären Aufnahmen in Deutschland 2010–2019 (04.01.2025). Im Internet: https://www.aerzteblatt.de/archiv/225759/Inanspruchnahme-der-ambulanten-Notfallversorgung-und-der-ungeplanten-stationaeren-Aufnahmen-in-Deutschland-2010-2019; Stand: 04.01.2025.

[18] Andrews H, Kass L. Non-urgent use of emergency departments: populations most likely to overestimate illness severity. Intern Emerg Med. 2018;13:893–900. 29380133 10.1007/s11739-018-1792-3

[19] Termin beim Psychotherapeuten. Kassenpatienten warten und warten und warten… Im Internet: https://www.stern.de/gesundheit/termin-beim-psychotherapeuten--kassenpatienten-warten-im-schnitt-fuenf-monate-7937794.html; Stand: Accessed 17 Sepember 2023.

[20] Hayes AF, Cai L. Using heteroskedasticity-consistent standard error estimators in OLS regression: an introduction and software implementation. Behav Res Methods. 2007;39:709–22. 18183883 10.3758/bf03192961

[21] Mangiapane S, Czihal T, Stillfried von. *D*. Entwicklung der ambulanten Notfallversorgung in Deutschland von 2009 bis 2020. Berlin; 2021.

[22] O’Reilly D, O’Neill C, et al. Mental health service provision and use among adults: an analysis of routine data. Br J Psychiatry. 2019;214(6):321–6.

[23] Walker ER, McGee RE, Druss BG. Mortality in mental disorders and global disease burden implications: a systematic review and meta-analysis. JAMA Psychiatr. 2015;72:334–41. 10.1001/jamapsychiatry.2014.2502 PMC4461039 25671328

[24] Osterloh F. 18.04. Reform der Notfallversorgung: Ein neuer Aufschlag – Deutsches Ärzteblatt (2024). Im Internet: https://www.aerzteblatt.de/archiv/reform-der-notfallversorgung-ein-neuer-aufschlag-42d77a4f-bc89-4fe4-b799-935c9ce93e94; Stand: 17.06.2025.

[25] Kirchner H, Ullrich H, Neu P et al. The significance of nonurgent psychiatric emergencies in an ED: a retrospective study. BMC Emerg Med 2023; 23: 131. 37940880 10.1186/s12873-023-00900-z PMC10631003

[26] Naouri D, Ranchon G, Vuagnat A, et al. Factors associated with inappropriate use of emergency departments: findings from a cross-sectional national study in France. BMJ Qual Saf. 2020;29:449–64. 31666304 10.1136/bmjqs-2019-009396 PMC7323738

[27] Scherer M, Lühmann D, Kazek A, et al. Patients attending emergency departments. Deutsches Arzteblatt Int. 2017;114:645–52. 10.3238/arztebl.2017.0645 PMC5651827 29034865

[28] Schmiedhofer M, Möckel M, Slagman M, Frick J, Ruhla S, Searle J. Patient motives behind low-acuity visits to the emergency department in Germany: a qualitative study comparing urban and rural sites. Im Internet: https://scholar.google.de/scholar?hl=de&as_sdt=0%2C5&q=schmiedhofer+2016+low-acuity&btnG=&oq=schmiedhofer+2016; Stand: Accessed 17 September 2023. 10.1136/bmjopen-2016-013323 PMC5129074 27852722

[29] Balestrieri M, Rucci P, Amendola D, et al. Emergency psychiatric consultations during and after the COVID-19 lockdown in italy. A multicentre study. Front Psychiatry. 2021;12:697058. 34211413 10.3389/fpsyt.2021.697058 PMC8239213

[30] Aly L, Sondergeld R, Hölzle P et al. Die COVID-19-Pandemie veränderte nicht die zahl, Aber die Art psychiatrischer notfälle: versorgungsdaten Aus vergleichszeiträumen von 2019 und 2020. Nervenarzt 2020; 91: 1047–9. 32710149 10.1007/s00115-020-00973-2 PMC7378304

[31] Schreiber S, Tene O, Mordel C, et al. A decrease in psychiatric consultations at the emergency room and inpatient wards of a large general hospital in Israel during the SARS-CoV-2 (COVID-19) pandemic. Gen Hosp Psychiatry. 2021;70:145–6. 33653611 10.1016/j.genhosppsych.2021.02.005 PMC7903901

[32] Ambrosetti J, Macheret L, Folliet A, et al. Impact of the COVID-19 pandemic on psychiatric admissions to a large Swiss emergency department: an observational study. Int J Environ Res Public Health. 2021. 10.3390/ijerph18031174. 33525740 10.3390/ijerph18031174 PMC7908206

[33] Pignon B, Gourevitch R, Tebeka S, et al. Dramatic reduction of psychiatric emergency consultations during lockdown linked to COVID-19 in Paris and suburbs. J Neuropsychiatry Clin Neurosci. 2020;74:557–9. 10.1111/pcn.13104 PMC7361336 32609417

[34] Kirchner H, Kirchner-Overfeld E-C, Juckel G et al. Drastischer Anstieg Von Fallzahlen Erfordert Umdenken Pflegez 2018; 71: 52–3.

[35] Thom J, Jonas B, Reitzle L et al. Trends in the diagnostic prevalence of mental disorders, 2012–2022. Deutsches Arzteblatt Int 2024; 121: 355–62. 10.3238/arztebl.m2024.0052 PMC11539879 38686592

[36] *Deutscher Ärzteverlag GmbH, Redaktion Deutsches Ärzteblatt*. Aktuelle Zahlen zum Suchtmittelkonsum (13.01.2025). Im Internet: https://www.aerzteblatt.de/nachrichten/105623/Aktuelle-Zahlen-zum-Suchtmittelkonsum; Stand: 13.01.2025.

[37] *Europäischer Rat*. Psychische Gesundheit-Arbeiten im Rat zum Thema psychische Gesundheit (13.01.2025). Im Internet: https://www.consilium.europa.eu/de/policies/mental-health/; Stand: 13.01.2025.

[38] Hoch O., Schwarzkopf, Gomes de Matos, Schneider. Cannabis – Zahlen und Fakten. In: Publishers PS, Hrsg. DHS Jahrbuch Sucht 2024. 1. Aufl. Lengerich: Pabst Science Publishers. 2024; 87.

[39] Myran DT, Pugliese M, McDonald AJ, et al. Cannabis use disorder emergency department visits and hospitalizations and 5-Year mortality. JAMA Netw Open. 2025;8:e2457852. 39913138 10.1001/jamanetworkopen.2024.57852 PMC11803479

[40] Zhu H, Wu L-T. Trends and correlates of Cannabis-involved emergency department visits: 2004 to 2011. J Addict Med 2016; 10: 429–36. 27574753 10.1097/ADM.0000000000000256 PMC5083207

[41] Roehler DR, Hoots BE, Holland KM, et al. Trends and characteristics of cannabis-associated emergency department visits in the united states, 2006–2018. Drug Alcohol Depend. 2022;232:109288. 35033959 10.1016/j.drugalcdep.2022.109288 PMC9885359

[42] Publishers PS, editor. DHS Jahrbuch Sucht 2024. 1. Aufl. Lengerich: Pabst Science Publishers. 2024.

[43] Chakravarthy B, Hoonpongsimanont W, Anderson CL et al. Depression, suicidal ideation, and suicidal attempt presenting to the emergency department: differences between these cohorts. Western J Emerg Med 2014; 15: 211–6. 10.5811/westjem.2013.11.13172 PMC3966461 24672614

[44] Kuo DC, Tran M, Shah AA et al. Depression and the suicidal patient. Emerg Med Clin North Am 2015; 33: 765–78. 26493522 10.1016/j.emc.2015.07.005

[45] Chao S. Overview of depression. Emerg Med Clin North Am. 2024;42:105–13. 37977742 10.1016/j.emc.2023.06.013

[46] Internetredaktion RBL. Depression – Internationale Studie bringt Licht in die Ursachen der Lebensfinsternis - DLR Gesundheitsforschung (13.01.2025). Im Internet: https://www.gesundheitsforschung-bmbf.de/de/depression-internationale-studie-bringt-licht-in-die-ursachen-der-lebensfinsternis-8288.php; Stand: 13.01.2025.

[47] Marchesi C, Brusamonti E, Borghi C, et al. Anxiety and depressive disorders in an emergency department ward of a general hospital: a control study. Emerg Med J. 2004;21:175–9. 14988342 10.1136/emj.2003.006957 PMC1726265

[48] Larkin GL, Beautrais AL, Spirito A et al. Mental health and emergency medicine: a research agenda. Acad Emerg Medicine: Official J Soc Acad Emerg Med 2009; 16: 1110–9. 10.1111/j.1553-2712.2009.00545.x PMC3679662 20053230

[49] Parkman T, Neale J, Day E et al. Qualitative exploration of why people repeatedly attend emergency departments for alcohol-related reasons. BMC Health Serv Res 2017; 17: 140. 28209195 10.1186/s12913-017-2091-9 PMC5314470

[50] Abar B, Hong S, Aaserude E et al. Access to care and depression among emergency department patients. J Emerg Med 2017; 53: 30–7. 28007366 10.1016/j.jemermed.2016.11.029 PMC5476517

[51] Long J, Knowles E, Bishop-Edwards L, et al. Understanding young adults’ reasons for seeking ‘clinically unnecessary’ urgent and emergency care: A qualitative interview study. Health Expect. 2021;24:1535–44. 34118177 10.1111/hex.13301 PMC8369113

[52] Ballou S, Mitsuhashi S, Sankin LS et al. Emergency department visits for depression in the united States from 2006 to 2014. Gen Hosp Psychiatry 2019; 59: 14–9. 31078012 10.1016/j.genhosppsych.2019.04.015

[53] Hill T, Jiang Y, Friese CR et al. Analysis of emergency department visits for all reasons by adults with depression in the united States. BMC Emerg Med 2020; 20: 51. 32571223 10.1186/s12873-020-00347-6 PMC7310062

[54] Radtke R. Weltweiter Anteil der bevölkerung, der unter angststörungen leidet, in Den Jahren 1990 Bis 2021 (13.01.2025). Im Internet: https://de.statista.com/statistik/daten/studie/1078851/umfrage/anteil-der-weltbevoelkerung-mit-angststoerungen/; Stand: 13.01.2025.

[55] Kirchner H, Kirchner-Overfeld E-C, Juckel G, et al. Häufigkeitsentwicklung alkoholassoziierter Vorstellungen in einer interdisziplinären großstädtischen Notaufnahme: Vergleich 2009 vs. 2014. SUCHT. 2018;64:109–16.

[56] Biancosino B, Vanni A, Marmai L et al. Factors related to admission of psychiatric patients to medical wards from the general hospital emergency department: a 3-year study of urgent psychiatric consultations. Int J Psychiatry Med 2009; 39: 133–46. 19860072 10.2190/PM.39.2.b

[57] Benarous X, Milhiet V, Oppetit A et al. Changes in the use of emergency care for the youth with mental health problems over decades: A repeated cross sectional study. Front Psychiatry 2019; 10: 26. 30787886 10.3389/fpsyt.2019.00026 PMC6372506

[58] Boyer L, Henry J-M, Samuelian J-C, et al. Mental Disorders among Children and Adolescents Admitted to a French Psychiatric Emergency Service. Emerg Med Int. 2013;2013:651530. 23431454 10.1155/2013/651530 PMC3568896

[59] Hegerl U, Althaus D, Schmidtke A et al. The alliance against depression: 2-year evaluation of a community-based intervention to reduce suicidality. Psychol Med 2006; 36: 1225–33. 16707028 10.1017/S003329170600780X

